# Cardiometabolic health, visceral fat and circulating irisin levels: results from a real-world weight loss study

**DOI:** 10.1007/s40618-020-01415-1

**Published:** 2020-09-06

**Authors:** T. Miazgowski, A. Kaczmarkiewicz, B. Miazgowski, J. Kopeć

**Affiliations:** 1 Department of Propedeutics of Internal Diseases and Arterial Hypertension, Pomeranian Medical University in Szczecin, ul. Unii Lubelskiej 1, 71-252 Szczecin, Poland; 2 Pomeranian Medical University in Szczecin, Szczecin, Poland; 3 Division of Epidemiology, Biostatistics and Public Health Practice, University of British Columbia, Vancouver, BC Canada

**Keywords:** Cardiometabolic health, Weight loss, Irisin, Real-world study

## Abstract

**Background:**

The aim of this pragmatic intervention study was to investigate changes in cardiometabolic outcomes, irisin plasma concentration, and body composition during a 4-month intervention in unselected obese individuals.

**Materials and methods:**

In 111 obese women aged 36.73 ± 7.2 years, we measured changes in weight, lipid profiles, glucose, insulin, Homeostatic Model Assessment-Insulin Resistance Index (HOMA-IR), uric acid, aminotransferases, and irisin. Body composition including lean mass (LM) and total (TF), gynoid (GF), android (AF), and visceral fat (VF) was assessed using densitometry. Physical activity was assessed using the International Physical Activity Questionnaire (IPAQ). The participants received tailored written advice targeting lifestyle according to current guidelines. At follow-up, patients rated their adherence in the self-administered questionnaire.

**Results:**

Mean weight loss in the whole group was 3.12 kg (− 3.3%); 26% of the women achieved the desired target of weight loss (> 5% of the initial weight), whereas weight decreased moderately in 50% and increased in 14%. In 86 women with weight loss, there were significant changes in HOMA-IR (− 13.8%), insulin (− 11.2%), alanine aminotransferase (− 8.0%), VF (− 7.0%), AF (− 5.4%), TF (− 4.7%), GF (− 2.8%) and LM (− 1.5%), whereas irisin and HDL-C levels and the mean IPAQ score did not change.

**Conclusions:**

In this real-world evidence study, a successful weight loss achieved only 26% of patients, with overall much better adherence to diet restriction than to exercise. However, even mild to moderate weight loss resulted in significant improvements in cardiometabolic health. Weight loss was associated with a modest LM decrease but did not influence plasma irisin.

## Introduction

It has been well-documented that weight loss in obese individuals lowers cardiometabolic risks, reduces the prevalence and burden of obesity-related comorbidities, and improves overall health. These beneficial changes are driven mainly by changes in body composition: reduction of fat mass and its redistribution. Current guidelines define the desired target as at least 5% weight reduction within the first 6 months of intervention [1, 2]. However, the guidelines are based mainly on data from reference studies performed in selected populations, in which dietary regimens and the level of exercising are usually pre-defined and strictly monitored during the intervention. Much less is known about how effective weight management techniques are in clinical practice. In real-world settings, weight loss frequently does not meet recommended targets [3, 4] and hence, metabolic benefits resulting from weight loss may be less apparent.

Irisin―an exercise-induced myokine produced by proteolytic cleavage of fibronectin type III domain-containing protein 5 (FNDC5)―is involved in the regulation of energy metabolism by activating thermogenesis [5]. In animal models, exogenous irisin showed an anorexigenic effect in rodents suggesting its inhibitory role in the central control of food intake [6, 7]. In addition, the myokine promoted β-cell survival and insulin secretion [8]. Studies of other effects of this myokine in humans have yielded conflicting results. Some but not all reports suggested a possible role of irisin in the modulation of glucose [9, 10] and lipid metabolism [11, 12] as well as its correlation with weight, surrogate measures of adiposity such as BMI and waist circumference (both negative [14] and positive [15]), and level of physical activity [16]. Based on these suggestions we hypothesized that in obese individuals, life style improvement and weight reduction might influence plasma levels of this myokine.

The aim of this pragmatic intervention study, based on a single-mode lifestyle counselling common in routine clinical practice, was to investigate changes in cardiometabolic outcomes, irisin plasma concentration, and body composition during a 4-month intervention in unselected obese individuals. Among the body composition parameters, we focused on visceral fat ―a key contributor to unfavorable changes in glucose and lipid metabolism, as well as lean mass, whose reduction has been suggested to be a detrimental effect of weight loss programs [17, 18].

## Materials and methods

### Study participants

Study participants were recruited from obese patients consecutively admitted for the first time to our outpatient secondary care unit for the treatment of obesity and metabolic disorders over a 1-year period. Patients with coexisting hypertension, dyslipidemia, hyperuricemia, type 2 diabetes, arthritis, and hypothyroidism received standard treatments. None of the patients received insulin, glucocorticoids, or protein supplements. In individual cases, patients took selenium, zinc, vitamin D, and herbal supplements. The study complied with all applicable institutional regulations regarding the ethical use of human volunteers in research and the terms of the Declaration of Helsinki. The Pomeranian Medical University Ethics Committee approved the study protocol, and all participants gave their written consent.

The study protocol included two visits: baseline and follow-up after 4–5 months. At the baseline visit, during an approximately 2-h session with a doctor and a trained dietician, participants were encouraged to implement healthier behaviors and received written advice targeting lifestyle modification according to current guidelines [1, 2]. Total daily energy requirements were calculated from basal metabolic rates and levels of physical activity (PA), and then reduced by 500–800 kcal per day. Weekly menus were prepared using a dietary calculator ALIANT (available at: https://aliant.com.pl), which includes recommended dietary allowance of micro- and macro elements. Moderate or moderate-to-vigorous PA for at least 30 min a day three or more days per week was recommended [19]. Both dietary regimen and the level of PA were tailored to patients’ individual habits, preferences, and needs. During both visits, anthropometric indices, laboratory tests, body composition, and physical activity were assessed.

### Anthropometric and biochemical measures

We measured height (at the baseline visit only) and weight, waist, hip, and neck circumferences (at both visits). Biochemical analyses encompassed fasting glucose, insulin, lipid profiles (triglycerides, total cholesterol and its LDL-C and HDL-C fractions), uric acid, aminotransferases (alanine; ALT and asparagine; AST), and irisin. Plasma irisin was assessed by ELISA using recombinant antibodies (Irisin Recombinant Human, Mouse, Rat, Canine; Phoenix Pharmaceuticals Inc., USA; catalog No. EK-067-29; the intra- and inter-assay variations were < 10% and < 15%, respectively; normal range provided by the manufacturer: 5.8–23.2 ng/ml). From fasting insulin and glucose, a Homeostatic Model Assessment-Insulin Resistance Index (HOMA-IR) was calculated, assuming a value of HOMA-IR < 2.5 as normal. We also calculated β-cell function (HOMA-%B). HOMA-IR and HOMA-%B were calculated using the HOMA Calculator v2.2.3 available at www.dtu.ox.ac.uk.ac.uk. Concomitant arterial hypertension, prediabetes/diabetes type 2, and abnormal values of waist circumference, blood lipids, HOMA-IR, uric acid, and ALT were considered to be cardiometabolic risk factors (CMRFs).

### Body composition

Body composition was assessed using dual-energy X-ray absorptiometry (DXA) (GE Healthcare Lunar Prodigy Advance; Madison, WI, USA) using the automatic whole-body scan mode. We analyzed total (TF), android (AF), and gynoid (GF) fat, as well as lean mass (LM), which in DXA is a surrogate measure of muscle mass. Visceral fat (VF) was computed using a GE Healthcare DXA device with the dedicated CoreScan® application.

### Physical activity

The International Physical Activity Questionnaire (IPAQ) was used to assess the self-reported level of habitual PA. The IPAQ questionnaire comprises seven questions related to different types of PA undertaken in a period of 7 days prior to completing the questionnaire. Total PA was categorized as low (< 600 METs min/week), moderate (600–3000 METs min/week), or vigorous (> 3000 METs min/week) [20].

### Self-assessment of adherence

At follow-up, patients were asked to what extent they adhered to the weight loss intervention, with three possible responses: ‘rather not’, ‘rather yes’ and ‘definitely yes’.

### Statistical analyses

Descriptive statistics included mean ± standard deviation (SD) for continuous variables and frequency distributions for categorical variables. Variables with a normal distribution were compared using paired Student’s *t* tests; otherwise, non-parametric Mann–Whitney *U*-tests and Kruskal–Wallis tests were used. Differences in study outcomes between patients with successful and modest weight loss, and without weight loss were compared using ANOVA; p for trend across categories was calculated using Cochran-Armitage test for trend. Correlations between pairs of quantitative variables were assessed using Pearson’s linear correlations or Spearman’s rho correlations for normally and non-normally distributed variables, respectively. The associations of body composition (predictors) with CMRFs (outcomes) were assessed using multiple linear regression models adjusted for BMI, age, and lipid-lowering, hypoglycemic, hypouricemic, and antihypertensive treatments. Due to multicollinearity among DXA-derived body composition parameters (the variance inflation factor > 10), predictors of weight loss were calculated using ridge regression [21]. The *λ* value (the Lagrange multiplier which controls the strength of the penalty term and minimizes mean square error) of 37.41 was included in the final model. The results of regression analyses were expressed as coefficient estimates (*β*^R^).

## Results

### Baseline characteristics

The study sample included 111 obese women aged 36.73 ± 7.2 years (range: from 19 to 49 years). At the baseline visit (Table 1), patients were taking medications for dyslipidemia (65%), hyperuricemia (27%), hypertension (23%), and type 2 diabetes (2%). Baseline laboratory tests revealed a high frequency of elevated HOMA-IR (69%), LDL-C (63%), total cholesterol (42%), and low HDL-C (37%). Hypertriglyceridemia and elevated ALT activity were also frequent. In patients with comorbidities, routine pharmacological treatments remained unchanged during the entire study period.

**Table 1:** Baseline characteristics of study participants

	*N*	%
Data from medical history		
Arterial hypertension	26	23
Type 2 diabetes	3	3
Dyslipidemia	72	65
Gallbladder diseases	15	14
Arthritis	2	2
Hyperuricemia	30	27
Hypothyroidism	9	8
Baseline laboratory findings		
Fasting glucose > 125 mg/dl	1	1
Fasting glucose 100–125 mg/dl	18	16
HOMA-IR ≥ 2.5	76	69
Total cholesterol > 200 mg/dl	47	42
LDL-C ≥ 115 mg/dl	70	63
HDL-C ≤ 50 mg/dl	41	37
Triglycerides ≥ 150 mg/dl	29	26
Uric acid > 6 mg/dl	22	20
ALT > 35 U/l	20	18
AST > 32 U/l	8	7

### Follow-up

Follow-up was performed after a median of 4.0 months (range: from 3.1 to 5.2 months). The mean weight loss in the whole group was 3.12 ± 1.2 kg (− 3.3%). However, only 29 women (26%) achieved the desired target of weight loss (5% or greater of the initial weight), whereas weight decreased moderately in 50% of the women and increased in 14% (Table 2). In the self-administered questionnaire, 43 patients (39%; mean weight change -6.6%; 95% CI: − 8.9% to − 4.2%) rated their adherence to lifestyle modification as ‘definitely yes’, 56 (50%; weight change − 2.4%; − 5.1% to 0.3%) as ‘rather yes’, and 12 (11%) as ‘rather not’ (all with weight gain).

**Table 2:** Distribution of weight change

	*n*	Weight change (kg)	Range (kg)
All	111 (100%)	− 3.12 ± 2.14	− 19.9 to 6.33
Referent (> 5%)	29 (26%)	− 7.34 ± 2.21	− 19.9 to − 5.19
Moderate (< 5%)	68 (61%)	− 3.01 ± 1.22	− 4.71 to − 0.72
No weight loss	14 (13%)	2.52 ± 1.99	0.36 to 6.33

Overall, 97 women (87.3%) lost weight by an average of 3.9 kg. As shown in Table 3, weight loss was accompanied by significant reductions in all anthropometric indices, results of laboratory tests, and body composition parameters. Among the latter, the greatest reductions were in VF mass (− 6%) and volume (− 7%). VF reduction was greater than reductions in TF, AF, and GF. Importantly, LM also slightly but significantly decreased (by 1.5%). At baseline, the majority of women declared moderate PA (600–3000 MET-min/week); however, a noticeable percentage (24%) rated it as low (< 600 MET-min/week). At follow-up the mean IPAQ score and the number of women with moderate or vigorous PA tended to increase; however, these changes did not reach statistical significance.

**Table 3:** Changes in anthropometric measures, laboratory tests, body composition, and physical activity between baseline and follow-up

	Baseline	Follow-up	% Change	*P*
Anthropometric measures				
Weight (kg)	95.23 ± 14.58	92.11 ± 14.17	− 3.3	< 0.001
	(70.0–139.8)	(67.4–135.2)		
Body mass index (kg/m^2^)	34.83 ± 4.27	33.75 ± 4.22	− 3.1	< 0.001
	(30.0–47.9)	(28.2–47.3)		
Waist (cm)	108.7 ± 10.79	104.9 ± 10.98	− 3.5	< 0.001
	(86.0–142.0)	(73.5–137.0)		
Hip (cm)	118.9 ± 9.86	115.32 ± 9.63	− 3.0	< 0.001
	(102.0–149.0)	(92.5–142.0)		
Neck (cm)	37.98 ± 2.71	37.20 ± 2.75	− 2.1	< 0.001
	(31.5–46.0)	(30.8–45.0)		
Laboratory tests				
Glucose (mg/dl)	92.05 ± 10.48	89.59 ± 9.43	− 2.7	< 0.001
	(69.4–138.5)	(68.1–117.2)		
Insulin (μIU/ml)	16.06 ± 9.33	14.26 ± 7.95	− 11.2	< 0.001
	(3.7–73.7)	(2.3–68.5)		
HOMA-IR	3.71 ± 2.39	3.20 ± 2.00	− 13.8	< 0.001
	(0.8–19.8)	(0.5–17.9)		
HOMA-%B	148.2 ± 36.7	142.7 ± 32.6	− 1.03	0.342
	(100.0–177.1)	(87.8–189.1)		
Total cholesterol (mg/dl)	191.51 ± 34.07	183.64 ± 31.48	− 4.1	< 0.001
	(119.4–298.2)	(112.9–281.9)		
LDL-C (mg/dl)	127.28 ± 31.88	121.35 ± 29.81	− 4.7	< 0.001
	(53.0–232.0)	(57.0–211.7)		
HDL-C (mg/dl)	54.53 ± 14.25	55.23 ± 13.45	1.3	0.189
	(30.0–98.8)	(30.4–100.0)		
Triglycerides (mg/dl)	133.96 ± 74.66	124.48 ± 65.55	− 7.1	< 0.001
	(42.4–434.0)	(39.5–404.5)		
ALT (U/l)	25.02 ± 15.54	23.01 ± 14.04	− 8.0	< 0.001
	(6.0–96.0)	(5.5–88.3)		
AST (U/l)	21.66 ± 11.89	20.37 ± 11.15	− 6.0	< 0.001
	(8.0–97.0)	(7.5–91.1)		
Uric acid (mg/dl)	5.17 ± 1.02	4.97 ± 0.91	− 3.9	< 0.001
	(3.2–8.4)	(3.4–8.0)		
Irisin (ng/ml)	12.85 ± 17.31	11.63 ± 12.33	− 9.5	0.193
	(5.9–136.1)	(5.5–117.0)		
Body composition				
Total fat (kg)	43.86 ± 10.16	41.82 ± 10.10	− 4.7	< 0.001
	(25.4–75.1)	(24.2–71.7)		
Total fat (%)	47.19 ± 4.97	45.88 ± 5.26	− 2.8	< 0.001
	(35.4–59.2)	(30.6–59.4)		
Android fat (kg)	7.60 ± 1.47	7.19 ± 1.38	− 5.4	< 0.001
	(5.1–11.7)	(4.8–10.6)		
Gynoid fat (kg)	15.16 ± 2.52	14.74 ± 2.48	− 2.8	< 0.001
	(10.5–22.0)	(10.2–22.7)		
Visceral fat (kg)	1.36 ± 0.57	1.28 ± 0.54	− 5.9	< 0.001
	(0.2–2.9)	(0.2–2.7)		
Visceral fat (cm^3^)	1446 ± 600	1345 ± 577	− 7.0	< 0.001
	(220–3035)	(205–2821)		
Lean mass (kg)	50.79 ± 5.76	50.03 ± 5.43	− 1.5	0.01
	(38.5–68.7)	(38.1–65.1)		
Physical activity (IPAQ)				
Total (MET-min/week)	3840 ± 1983	4165 ± 1909	8.4	0.212
	(310–6100)	(420–6190)		
< 600 (MET-min/week) (n)	26 (24%)	20 (18%)	− 6.0	0.320
600–3000 (MET-min/week) (n)	48 (43%)	52 (47%)	4.2	0.586
> 3000 (MET-min/week) ( n)	37 (33%)	39 (35%)	1.9	0.777

Data are means ± SD (range) or the number of cases (%)

In ridge regression, the magnitude of weight loss was associated with reductions in TF (*β*^R^ = 15.8; *p* < 0.001), AF (*β*^R^ = 9.01; *p* = 0.003) and GF (*β*^R^ = 8.43; *p* = 0.021), whereas associations with VF mass and LM were not significant.

As summarized in Table 4, surrogate measures of adiposity (BMI, neck, waist, and hip circumference) tended to correlate with CMRFs. Neck circumference showed the strongest correlations with CMRFs, particularly with HOMA-IR, insulin, glucose, HDL-C, and uric acid. Neck circumference was positively correlated with waist (*r* = 0.590) and hip circumference (*r* = 0.378). In contrast to other anthropometric measures, neck circumference also correlated with LDL-C and triglycerides.

**Table 4:** Correlations between study variables (baseline and follow-up data combined)

	Uric acid (mg/dl)	Glucose (mg/dl)	Insulin (μIU/ml)	HOMA-IR	HDL-C (mg/dl)	LDL-C (mg/dl)	Triglycerides (mg/dl)	ALT (U/l)	IPAQ (METs)	Irisin (ng/ml)
Weight (kg)	0.208 ^**a**^	0.013	0.268 ^**b**^	0.212 ^**a**^	− 0.166	− 0.109	0.033	0.245 ^b^	− 0.133	0.192 ^a^
BMI (kg/m^2^)	0.216 ^b^	0.099	0.292 ^b^	0.221 ^b^	− 0.162 ^a^	− 0.002	0.027	0.234 ^b^	− 0.051	0.188 ^a^
Neck girth (cm)	0.302 ^b^	0.177 ^a^	0.401 ^b^	0.373 ^b^	− 0.346 ^b^	0.162 ^a^	0.310 ^b^	0.337 ^b^	− 0.121	0.196 ^a^
Waist girth (cm)	0.223 ^b^	0.141 ^a^	0.306 ^b^	0.212 ^b^	− 0.156 ^a^	− 0.016	0.063	0.331 ^b^	− 0.005	0.194 ^a^
Hip girth (cm)	0.152 ^a^	0.041	0.198 ^b^	0.154 ^a^	− 0.095	− 0.113	0.003	0.147 ^a^	− 0.125	− 0.092
Total fat (kg)	0.161 ^a^	0.047	0.209 ^b^	0.167 ^a^	− 0.092	− 0.070	0.003	0.186 ^b^	− 0.112	0.112
Visceral fat (kg)	0.362 ^b^	0.165 ^a^	0.279 ^b^	0.240 ^b^	− 0.207 ^b^	0.132	0.200 ^b^	0.344 ^b^	− 0.061	0.095
Android fat (kg)	0.239 ^b^	0.090	0.168 ^a^	0.158 ^a^	− 0.137 ^a^	− 0.023	0.101	0.283 ^b^	− 0.097	− 0.067
Gynoid fat (kg)	0.168	− 0.032	0.098	0.117	− 0.087	− 0.126	− 0.014	0.091	− 0.158 ^a^	0.207 ^a^
Lean mass (kg)	0.251 ^b^	− 0.018	0.180	0.174	− 0.209 ^b^	− 0.087	0.092	0.212 ^b^	0.159 ^a^	0.200 ^a^

^a^*p* < 0.05; ^b^*p* < 0.01. Correlation coefficients were calculated from baseline and follow-up data combined

Among DXA-derived body composition parameters, VF was a positive correlate of CMRFs, particularly glucose, HOMA-IR, triglycerides and uric acid, and these correlations were stronger than those of TF, AF and GF. Similarly, in multiple linear regression models adjusted for BMI, age, and lipid-lowering, hypoglycemic, hypouricemic and antihypertensive treatments, VF was positively associated with HOMA-IR (*β* = 0.42; *p* < 0.001) and uric acid (*β* = 0.39; *p* = 0.003), whereas the associations were weaker for TF (*β* = 0.30; *p* = 0.009 and *β* = 0.19; *p* = 0.041, respectively) and AF (*β* = 0.36; *p* = 0.004 and *β* = 0.31; *p* = 0.006, respectively), and non-significant for GF.

**Fig. 1:**
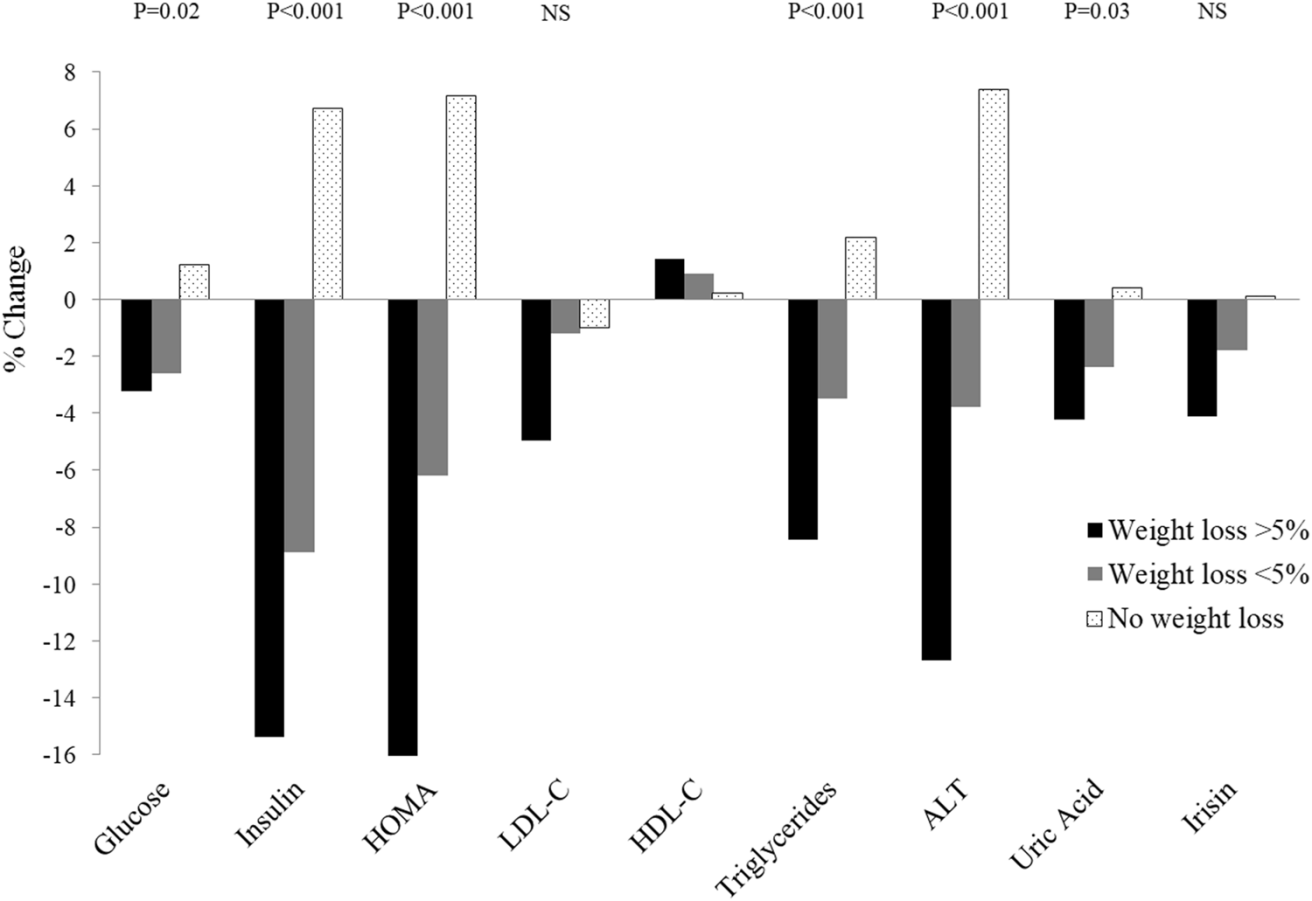
Changes in biochemical parameters by weight change. P-value refers to trend across categories

**Fig. 2:**
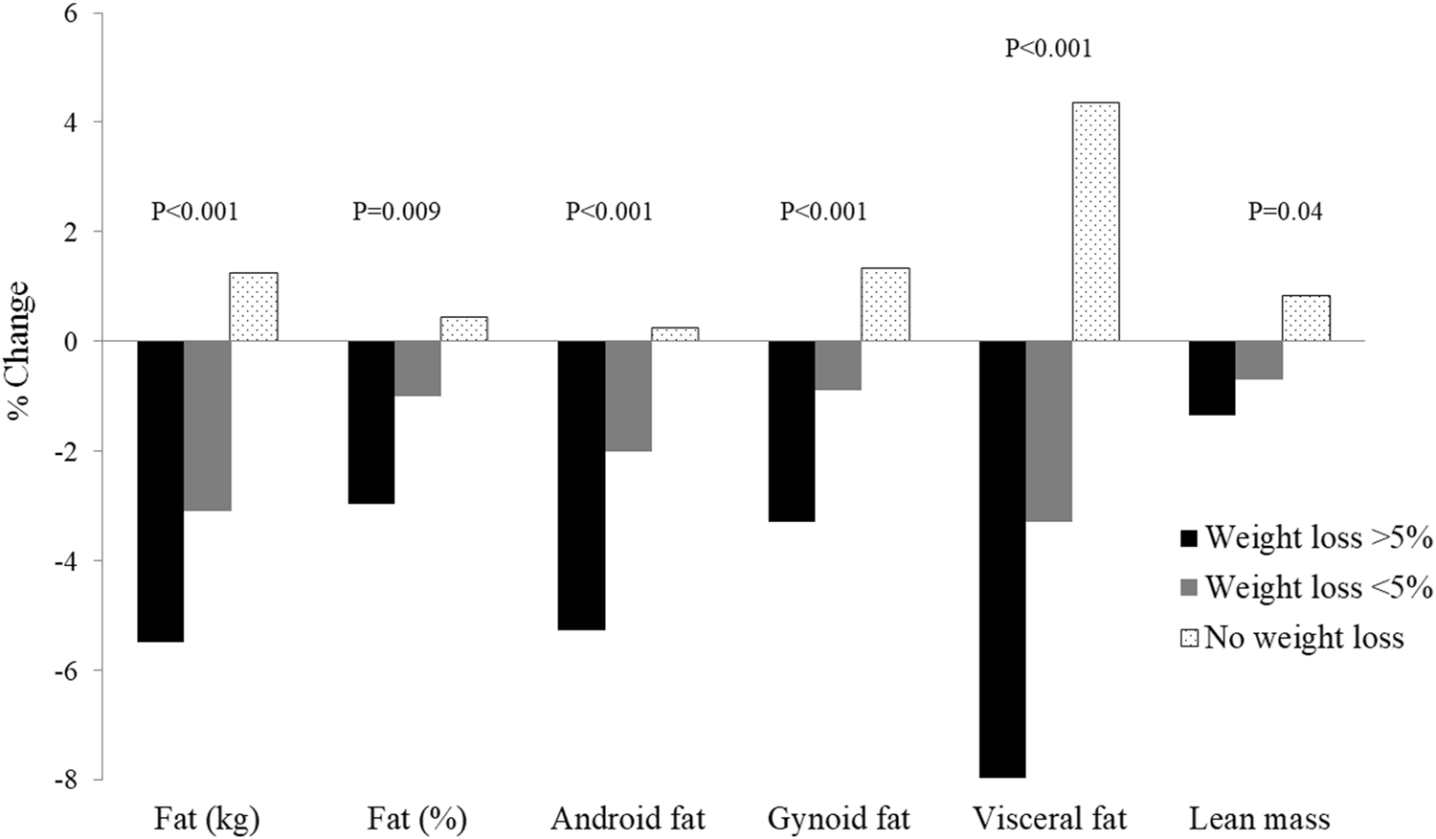
Changes in body composition by weight change. P-value refers to trend across categories

As expected, the beneficial changes in laboratory CMRFs (Fig. 1) and body composition (Fig. 2) significantly differed between groups with and without weight loss in a dose–response manner. In those without weight loss, insulin, HOMA-IR and ALT significantly increased (by 6.7%, 7.2%, and 7.1%, respectively; all *p* < 0.05 vs. baseline). Other obesity measures tended to increase, particularly VF (by 4.4% vs. baseline), but these changes were not significant. The mean IPAQ score did not differ between women with a weight reduction of at least 5% and those whose weight did not decrease during the study period.

### Irisin

Irisin plasma levels were within the reference range and did not differ between baseline and follow-up (Table 3). In the analysis of all results combined from samples collected at baseline and follow-up (Table 4), irisin showed weak but significant positive correlations with weight, BMI, neck and waist circumferences, LM, and GF. Irisin was also positively correlated with insulin (*r* = 0.202; *p* = 0.002) and HOMA-IR (*r* = 0.184; *p* = 0.006). Although irisin was weakly correlated with weight, changes in weight did not influence irisin levels (Fig. 1). No correlations were found between irisin and other parameters of body composition, blood lipids, and the IPAQ score.

## Discussion

In this real-world intervention that was based on a single-mode lifestyle counseling given to non-selected obese women, the desirable goal of weight loss > 5% of initial value was achieved by 26% of participants. As expected, cardiometabolic improvements from weight loss increased in a dose–response manner. However, even mild to moderate weight reduction in the order of 3–4%, as found in our study, was associated with measurable beneficial cardiometabolic effects, including significant reductions in anthropometric indices (BMI and waist, hip and neck circumference), glucose, total cholesterol, LDL-C, ALT (a biomarker of fatty liver disease [22]), triglycerides, aminotransferases, and uric acid. The greatest reductions were seen for insulin levels and HOMA-IR (− 11% and − 14%, respectively). Among the DXA-derived body composition parameters, VF was the strongest positive correlate of blood lipids, insulin resistance, and uric acid level.

Earlier studies of these associations were often hampered by insufficient methodology for quantifying visceral adipose tissue content, specifically, using indirect VF measurements such as sagittal abdominal diameter, waist circumference or waist-to-hip-ratio. Imaging techniques such as computed tomography or magnetic resonance imaging (MRI) accurately quantify VAT but these techniques are costly, time-consuming, or associated with a risk of radiation. We report here for the first time the association of VF and serum uric acid level using quantification of VF by DXA. This modality measures VF with high precision and has demonstrated a strong correlation with VF measured by computed tomography and MRI [23]. Similarly to our results, Rospleszcz et al. [24] recently found an association of VF with uric acid (*β* = 0.55) in overweight individuals using MRI. Altogether, these findings confirm that VF mass influences not only insulin sensitivity, lipid profiles, and function of the liver, but also purine metabolism.

Although it has been demonstrated that exercise can play a role in both short- and long-term weight loss and weight maintenance [1, 19], in routine practice it is often challenging for patients to engage in a regular exercise program and to continue that program as a lifestyle modification [25]. This issue also pertains to our study. Although we tailored exercise recommendations to an individual’s preferences and habitual PA, adherence to exercise was relatively weak. The mean IPAQ score and the number of women with moderate to vigorous PA tended to increase, but this trend was not statistically significant. In addition, IPAQ was not correlated with weight or changes in other anthropometric indices suggesting that PA played a minimal role in weight loss. These findings are supported by a recent report in which a hypocaloric diet without prescription of physical activity was sufficient to lose weight in the short term [26].

As expected, weight loss was associated with a reduction of TF and its two main compartments: AF and GF. However, the greatest reductions occurred in VF mass and volume. Although VF is a relatively small fat depot (in this study it comprised only 1.4% of body weight and 3% of TF), but due to its high lipolytic activity and ability to release inflammatory cytokines, it has been found to be a robust predictor of cardiovascular disease [27, 28]. Previous studies have demonstrated the effectiveness of non-pharmacological methods for VF reduction using diet alone or in combination with aerobic exercise or high-intensity interval training [26, 29–31]. Our results strongly suggest that lifestyle modification based on dietary restriction combined with modest PA can be an effective approach to reducing VF mass and volume.

Another finding of this study is the diminution of LM in weight loss programs. Following the intervention, we found LM to be reduced by approximately 0.75 kg (1.5% of the initial mass), but this reduction did not contribute substantially to overall weight loss. The value of this finding may be weakened by some methodological concerns regarding the assessment of LM by DXA. Although DXA-LM is mainly composed of muscles, LM is not a direct measure of muscle mass because this compartment contains also other tissues such as blood, skin, tendons and viscera, and the volume of all these tissues can be influenced by hydration status, which may change with age, exercise, weight loss and other factors. In addition, a recent study demonstrated a wide variation in the correlation between DXA and MRI (the reference modality) in the regional muscle mass quantification, from 0.26 in the trunk do 0.88 in legs [32]. Furthermore, from studies evaluating changes in structural features and composition of the skeletal muscle using computed tomography and MRI [33, 34] it can be assumed that weight gain and low PA lead to increases in intramuscular adipose tissue (IMAT) content, which infiltrates skeletal muscle fibers and is functionally considered an ectopic fat depot [35]. As DXA-LM does not quantify IMAT, we speculate that LM reduction in our study might be driven, at least partially, by a decrease in IMAT corresponding to changes in total and regional fat, and possibly also other ectopic fat depots. Consequently, a true muscle wasting effect would be negligible. Therefore, LM reduction is often only modest and of no substantial effect on overall weight loss. Such interpretation could be applied also to other studies reporting modest LM reductions in response to weight loss [36].

The relationship of plasma irisin concentration with chronic exercise training or habitual PA is controversial as both increasing [37] and decreasing [38] levels of this myokine have been observed. Likewise, contradictory evidence has been shown associating irisin levels with CMRFs, including blood lipids, anthropometric parameters, and fat mass [14, 15, 39]. In our study, circulating irisin was correlated with adiposity measures but, in general, these correlations were weak. We also found a slightly stronger correlation with insulin and HOMA-IR. On the other hand, similarly to Fukushima et al. [9], we did not find irisin to be associated with weight loss. To our knowledge, in only one study calorie restriction has been shown to decrease circulating irisin levels, with a subsequent increase if the weight was regained [13]. However, the population sampled in this study had a higher baseline BMI (35.8 kg/m^2^) and insulin concentration (13.6 (μIU/ml), and greater weight loss (-6.1 kg) over time. Importantly, they also used a different assay to measure irisin. The key point of explaining these discrepancies may be related to insulin sensitivity. Many studies, including our report of a positive associations of irisin with HOMA-IR [10, 39, 40], suggest that it is not body weight per se but rather the level of insulin resistance that predisposes the individual to a higher irisin concentration. However, it is unclear whether this phenomenon represents exclusively a compensatory increase of irisin to overcome an underlying irisin resistance in both obese and non-obese subjects.

Our results may have some methodological limitations. Firstly, some researchers have questioned the reliability of circulating irisin levels measured with commercial ELISA assays due to still unresolved issue of transcription of the irisin precursor FNDC5 gene across species, the high levels of inter- and intra-assay variation with these assays, and the large reported range of irisin levels (0.02–4300 ng/ml) [41]. Secondly, the accuracy of DXA-LM measurement can be affected by hydration status. To minimize this effect, all patients DXA scans were performed in the morning after an overnight fast. Thirdly, the results of body composition in this study may not be reproducible using a different DXA machine and software. Finally, patients in this study were taking medications for comorbidities that might affect the study outcomes, particularly biochemical CMRFs. However, all treatments were stable during the entire study period; therefore, they should not affect the reported changes in outcomes over time.

In conclusion, in this real-world evidence study, lifestyle modification in obese women resulted in successful (> 5%) weight loss in 26% of patients, with overall much better adherence to diet restriction than to exercise. However, even mild to moderate weight loss resulted in significant improvements in cardiometabolic health. Weight loss was associated with a decrease in VF mass and a modest LM decrease but did not influence plasma irisin levels.
